# Post-craniotomy Troponin Elevation Without Ischemia After Traumatic Brain Injury: A Case Report and Management Framework

**DOI:** 10.7759/cureus.106662

**Published:** 2026-04-08

**Authors:** Ana Gabriela Álvarez Barrientos, Kimberly Carvajal Méndez, Dennis Ulate Fuentes, Freddy Gutiérrez Rivera, Daniel Aragón Leiva

**Affiliations:** 1 Faculty of Medicine, University of Ibero-America (UNIBE), San José, CRI; 2 Faculty of Medicine, Universidad de Costa Rica, San José, CRI; 3 Faculty of Medicine, Universidad Latina de Costa Rica, San José, CRI

**Keywords:** acute coronary syndrome, craniotomy, myocardial injury, neurocardiac axis, takotsubo cardiomyopathy, traumatic brain injury, troponin elevation, type 2 myocardial infarction

## Abstract

Cardiac complications after neurosurgical procedures are uncommon but may present significant diagnostic and therapeutic challenges, particularly when anticoagulation carries a high risk of intracranial hemorrhage. We report the case of a 40-year-old woman with no prior medical history who sustained blunt craniofacial trauma from a heavy metallic object. Head CT revealed bilateral depressed frontal fractures with associated epidural hematomas and cerebral contusions. The patient underwent bilateral frontal craniotomy for hematoma evacuation and osteosynthesis. During the early postoperative period, she developed acute chest pain, marked tachycardia (up to 170 bpm), and a significant elevation in troponin levels (peak 244.7 ng/L). Serial electrocardiograms (ECGs) demonstrated sinus rhythm without dynamic ischemic changes, and echocardiography showed preserved biventricular function without regional wall motion abnormalities. Given the recent craniotomy and high bleeding risk, anticoagulation and antiplatelet therapy were deferred. The clinical presentation and diagnostic findings were consistent with acute myocardial injury likely secondary to hemodynamic stress and sympathetic overactivation rather than acute coronary syndrome. The patient improved with conservative management, achieving full recovery without neurological or cardiac complications.

This case highlights the importance of distinguishing neurogenic or stress-related myocardial injury from true ischemia in postoperative neurosurgical patients. Mechanisms such as catecholamine surge and paroxysmal sympathetic hyperactivity (neuro-storm) may contribute to transient myocardial injury in this setting. A multidisciplinary approach integrating ECG trends, biomarker kinetics, and echocardiography can support accurate diagnosis while minimizing bleeding risk. These findings should be interpreted in the context of a single case, and further studies are needed to better characterize this phenomenon.

## Introduction

Cardiac complications following neurosurgical procedures are uncommon but can result in significant morbidity. These cases require careful differentiation between true ischemic events and stress-related or neurogenic myocardial injury, particularly when anticoagulation may increase the risk of intracranial hemorrhage [1]. Troponin elevation has been reported in a significant proportion of patients with acute traumatic brain injury (TBI), with estimates ranging from 20% to 40%, and is often associated with worse clinical outcomes, even in the absence of primary cardiac pathology [2,3].

The neurocardiac axis describes the bidirectional interaction between the central nervous system and the heart, explaining how acute brain injury can trigger transient myocardial dysfunction, arrhythmias, or troponin elevation through sympathetic overactivation and catecholamine surge, a phenomenon often referred to as neurogenic myocardial stunning [2,3]. This spectrum includes entities such as acute myocardial injury (defined as troponin elevation without evidence of ischemia) and type 2 myocardial infarction (T2MI), the latter resulting from a myocardial oxygen supply-demand imbalance (e.g., severe tachycardia or hypotension) without acute plaque rupture. These conditions may overlap with stress-induced (Takotsubo) cardiomyopathy but differ in their pathophysiological drivers and management strategies.

In postoperative neurosurgical patients, the occurrence of chest pain and troponin elevation presents a critical diagnostic dilemma, as standard acute coronary syndrome (ACS) therapies, such as dual antiplatelet therapy or anticoagulation, can be catastrophic in the setting of recent craniotomy [1-3]. While the incidence of troponin elevation is well-documented in the acute phase of TBI, there is a lack of standardized management pathways for patients who develop these findings specifically in the immediate postoperative period after neurosurgical intervention.

We report the case of a postoperative craniotomy patient who developed transient troponin elevation consistent with stress-related myocardial injury and was successfully managed conservatively. This case addresses a gap in the existing literature on postoperative troponin elevation after craniotomy, particularly in the absence of electrocardiographic or echocardiographic evidence of ischemia by detailing the diagnostic challenges and proposing a structured, multidisciplinary approach to diagnosis and management in this high-risk population where invasive evaluation is often contraindicated. 

## Case presentation

**Figure 1:**
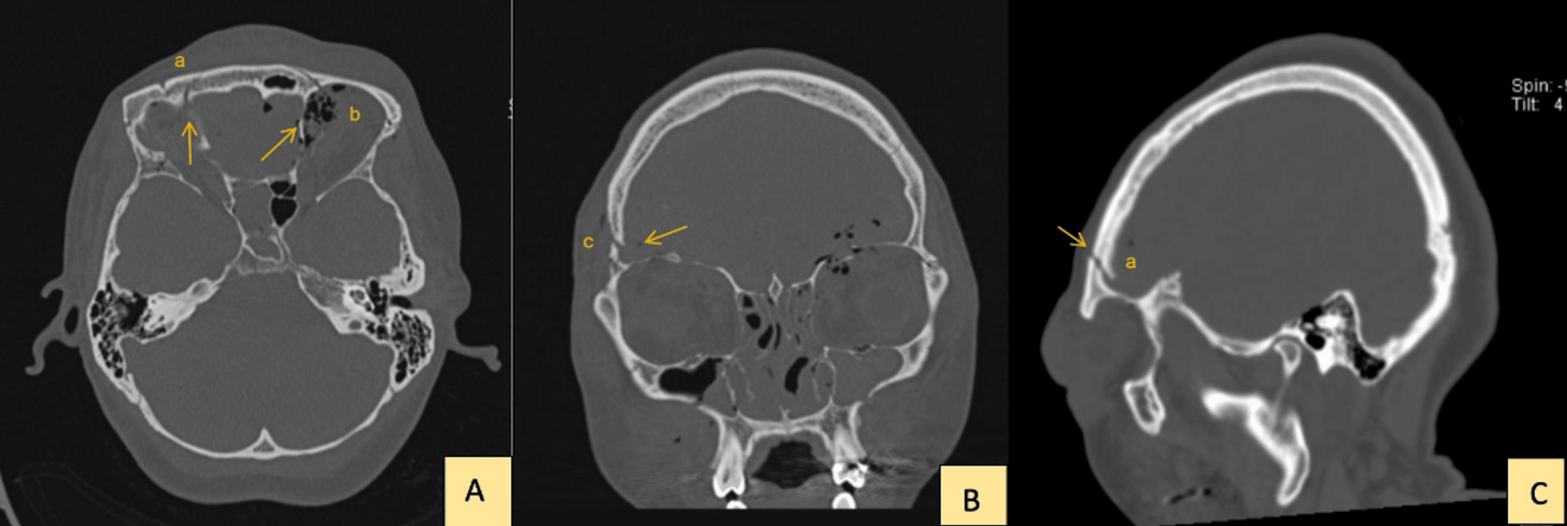
Craniofacial CT (bone window) showing bilateral frontal fractures with right-sided depression and associated orbital injuries. A (axial, non-contrast): Bilateral frontal bone fractures (a) with a depressed right frontal component and adjacent air locules/pneumocephalus (b) (arrows). B (coronal reformat): Comminuted fracture of the right lateral orbital wall extending to the orbital surface of the ipsilateral greater wing of the sphenoid (c) (arrow). C (sagittal reformat): Depressed right frontal fracture involving the orbital roof (a) (arrow).

A 40-year-old woman with no significant medical history sustained blunt craniofacial trauma from a heavy metallic object. Head computed tomography (CT) revealed a complex depressed frontal bone fracture involving the bilateral orbital roofs, bifrontal epidural hematomas, and hemorrhagic contusions in both frontal lobes. Additional findings included multifragmentary fractures of the lateral orbital wall, the orbital surface of the ipsilateral greater wing of the sphenoid, a comminuted fracture of the posterior third of the nasal septum, and a nondisplaced nasal bone fracture (Figure 1).

**Figure 2:**
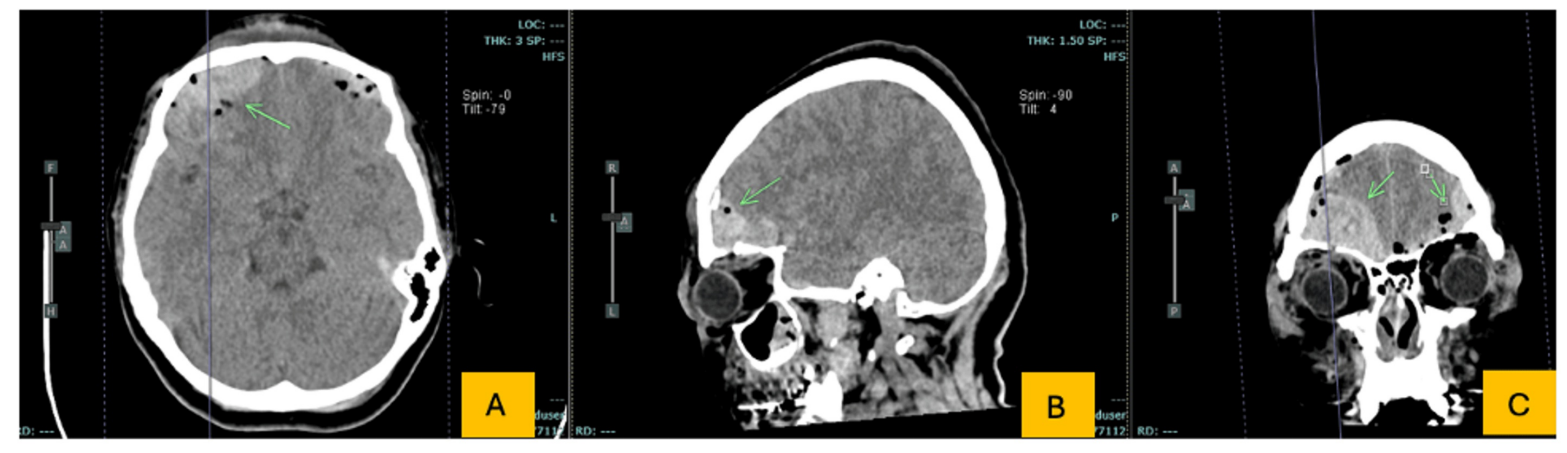
Head CT (brain window) showing bilateral frontal hemorrhagic contusions with traumatic pneumocephalus. A (axial, non-contrast): Patchy hyperdensities in the frontal lobes consistent with bilateral hemorrhagic contusions and scattered intracranial air locules (arrow). B (sagittal reformat): Intracranial air over the right frontal convexity compatible with traumatic pneumocephalus (arrow). C (coronal reformat): Bilateral frontal hemorrhagic contusions with multiple intracranial air locules (arrows).

The patient underwent bilateral frontal craniotomy for hematoma evacuation and osteosynthesis. Approximately six hours after surgery, during administration of postoperative intravenous maintenance fluids and analgesic medications, she developed acute oppressive chest pain radiating to the left arm and back, accompanied by dyspnea and marked tachycardia (heart rate 170 bpm). Sublingual nitroglycerin, administered for suspected ischemic chest pain, produced partial pain relief but precipitated marked hypotension (mean arterial pressure of 46 mmHg), highlighting the hemodynamic vulnerability in this postoperative setting. Symptoms resolved completely after prompt fluid resuscitation (Figure 2). 

Given the high postoperative bleeding risk, anticoagulation and dual antiplatelet therapy were withheld, and the patient was transferred to the intensive care unit (ICU) for close monitoring. Serial electrocardiograms (ECGs) demonstrated sinus tachycardia without dynamic ST-segment or T-wave changes suggestive of ischemia. High-sensitivity troponin peaked at 220.6 ng/L (reference range <3.8 ng/L) and declined to 20.8 ng/L within 36 hours, while NT-proBNP remained mildly elevated (Table 1). Echocardiography, performed at an outside institution, demonstrated preserved left ventricular ejection fraction (56%) with no regional wall motion abnormalities.

**Table 1:** Serial biomarker trends. Cardiac biomarkers demonstrated a transient elevation consistent with stress-related myocardial injury, followed by subsequent normalization. Reference range: High-sensitivity troponin <3.8 ng/L according to the institutional laboratory.

Timepoint	Troponin (ng/L)	NT-proBNP (pg/mL)	Notes
Baseline	<3.8	191.4	Pre-event
+6 hours	164.2	183.2	During episode
+12 hours	220.6	320	Peak
+24 hours	72.3	177	Downtrend
+36 hours	20.8	--	Normalization

Based on the absence of ischemic ECG changes, preserved ventricular function, and the transient pattern of biomarker elevation, ACS was considered unlikely. The clinical picture was most consistent with acute myocardial injury secondary to hemodynamic stress and sympathetic activation in the postoperative setting. Alternative diagnoses, including Takotsubo cardiomyopathy, pulmonary embolism, and coronary vasospasm, were considered less likely and were clinically excluded due to the absence of supporting clinical, electrocardiographic, or echocardiographic findings.

The patient remained hemodynamically stable thereafter, with gradual normalization of cardiac biomarkers and complete resolution of symptoms. Her postoperative course was otherwise uneventful, and she was discharged in stable condition without neurological or cardiac sequelae. Short-term follow-up revealed no recurrence of symptoms.

The original ECG tracings were not available for retrieval at the time of manuscript revision. Similarly, the echocardiographic study was performed at an outside institution, and only the written report documenting preserved left ventricular systolic function (ejection fraction 56%) without regional wall motion abnormalities was available for review.

## Discussion

Cardiovascular complications following TBI are increasingly recognized due to their complex pathophysiology and diagnostic challenges. Early myocardial injury has been reported in up to 20-30% of patients with moderate to severe TBI and is associated with increased in-hospital mortality [1]. The neurocardiac axis describes the bidirectional interaction between the central nervous system and the heart, whereby acute brain injury can trigger sympathetic overactivation, catecholamine surge, and hemodynamic instability, leading to myocardial injury in the absence of coronary obstruction [2,3].

An important contributor to this phenomenon is paroxysmal sympathetic hyperactivity (PSH) or “neurostorming”, which often follows severe TBI. PSH is characterized by episodic tachycardia, hypertension, hyperthermia and increased catecholamine release. This exaggerated adrenergic state can precipitate myocardial oxygen supply-demand mismatch (type 2 myocardial infarction) and direct catecholamine-mediated myocardial toxicity, contributing to transient troponin elevation. In our patient, the extreme tachycardia (170 bpm) and subsequent hypotension (MAP 46 mmHg) after nitroglycerin administration created a profound supply-demand imbalance, which is the hallmark of T2MI rather than acute plaque rupture (type 1 MI).

Our case reflects these mechanisms, with a transient elevation in troponin levels, absence of ischemic ECG changes, and preserved ventricular function on echocardiography, demonstrating a preserved ejection fraction (56%) without wall motion defects. These findings support a diagnosis of acute myocardial injury related to hemodynamic stress and sympathetic activation rather than ACS. Importantly, this presentation differs from Takotsubo cardiomyopathy, which typically demonstrates regional wall motion abnormalities (e.g., apical ballooning), and from type 1 myocardial infarction, which is associated with plaque rupture and coronary thrombosis. Instead, the observed pattern is more consistent with a type 2 myocardial injury due to supply-demand mismatch. Furthermore, other differentials such as pulmonary embolism were ruled out by the absence of right ventricular strain on echocardiography, and coronary vasospasm was considered unlikely given the rapid normalization of biomarkers and the clear presence of a physiological trigger (TBI and surgical stress).

Case reports by Wang et al. and Kumari et al. have described Takotsubo cardiomyopathy following TBI, mediated by catecholamine toxicity [2,3]. In contrast, our patient maintained preserved ventricular function and demonstrated rapid biomarker normalization, suggesting a milder and reversible form of neurogenic myocardial injury within the same pathophysiological spectrum.

Evanson et al. highlighted that extracranial complications, including cardiovascular dysfunction, can occur at any stage of TBI recovery [4]. Similarly, Velayudham et al. reported postoperative neurocardiac complications, such as complete heart block following craniotomy, emphasizing the importance of multidisciplinary management [5]. In the postoperative neurosurgical setting, additional contributors such as pain, hypovolemia, anesthetic agents, and vasoactive medications may further exacerbate sympathetic activation and myocardial stress.

According to the 2023 European Society of Cardiology (ESC) Guidelines for the Management of Acute Coronary Syndromes, postoperative troponin elevation should be interpreted cautiously and in clinical context, as many cases represent myocardial injury rather than infarction. The guideline emphasizes distinguishing type 2 ischemic imbalance or non-ischemic myocardial injury from type 1 myocardial infarction based on clinical presentation, ECG findings, and imaging, in order to avoid unnecessary antithrombotic or anticoagulant therapy when ischemia is unproven [6].

From a clinical perspective, this case highlights the importance of a structured and multidisciplinary diagnostic approach: (i) Baseline and serial high-sensitivity troponin to assess kinetics; (ii) 12-lead ECG to rule out dynamic ST-segment changes; and (iii) Early bedside echocardiography. In the absence of ischemic features, conservative management with close hemodynamic monitoring may be appropriate, particularly in patients with high bleeding risk, such as those in the immediate postoperative period after craniotomy. Advanced imaging modalities such as coronary CT angiography or cardiac magnetic resonance imaging may be considered in selected cases when diagnostic uncertainty persists, although they were not pursued in this case due to rapid clinical improvement, low suspicion for obstructive coronary disease and the high risk of transport in the early post-craniotomy phase.

Reviews on neurogenic myocardial injury and troponin elevation in critical illness confirm that non-ischemic etiologies are common and often transient [7,8]. However, even in the absence of overt ischemia, troponin elevation remains a prognostic marker in critically ill and postoperative patients [9]. Recent studies further support that in neurocritical care populations, troponin elevation frequently reflects stress-related myocardial dysfunction rather than infarction [10].

This case contributes to the existing literature by illustrating a reversible form of postoperative myocardial injury in a high-risk neurosurgical patient, in whom invasive management was safely avoided. It underscores the importance of integrating clinical, electrocardiographic, and imaging findings to guide individualized management. Given that this is a single case report, conclusions should be interpreted with caution. Further studies involving larger patient populations are needed to better characterize the incidence, mechanisms, and optimal management strategies of neurocardiac complications following TBI and neurosurgical procedures.

## Conclusions

Postoperative troponin elevation in neurosurgical patients does not necessarily indicate ACS. Instead, it often reflects acute myocardial injury or T2MI triggered by sympathetic overactivation, hemodynamic stress, or neurocardiac mechanisms such as paroxysmal sympathetic hyperactivity. Distinguishing these entities from true ischemic events is essential to avoid unnecessary antithrombotic therapy and minimize the risk of intracranial hemorrhage in the early postoperative phase.

This case highlights the utility of a structured, multidisciplinary approach integrating serial high-sensitivity troponin kinetics, ECG monitoring, and early bedside echocardiography. In high-risk patients with elevated biomarkers but no objective evidence of regional wall motion abnormalities or ischemic ECG changes, conservative management can be safe and effective. While based on a single case, these findings underscore the need for standardized management pathways and further prospective studies to better define the optimal management of myocardial injury in neurocritical care populations.
